# Psychometrics of the Short Form 36 Health Survey Version 2 (SF-36v2) and the Quality of Life Scale for Drug Addicts (QOL-DAv2.0) in Chinese Mainland Patients with Methadone Maintenance Treatment

**DOI:** 10.1371/journal.pone.0079828

**Published:** 2013-11-20

**Authors:** Kaina Zhou, Guihua Zhuang, Hongmei Zhang, Peifeng Liang, Juan Yin, Lingling Kou, Mengmeng Hao, Lijuan You

**Affiliations:** Department of Epidemiology and Biostatistics, School of Public Health, Xi’an Jiaotong University Health Science Center, Xi’an, Shaanxi, China; Catholic University of Sacred Heart of Rome, Italy

## Abstract

**Objective:**

To test psychometrics of the Short Form 36 Health Survey version 2 (SF-36v2) and the Quality of Life Scale for Drug Addicts (QOL-DAv2.0) in Chinese mainland patients with methadone maintenance treatment (MMT).

**Methods:**

A total of 1,212 patients were recruited from two MMT clinics in Xi’an, China. Reliability was estimated with Cronbach’s α and intra-class correlation (ICC). Convergent and discriminant validity was assessed using multitrait-multimethod correlation matrix. Sensitivity was measured with ANOVA and relative efficiency. Responsiveness was evaluated by pre-post paired-samples *t*-test and standardized response mean based on the patients’ health status changes following 6-month period.

**Results:**

Cronbach’s α of the SF-36v2 physical and mental summary components were 0.80 and 0.86 (eight scales range 0.73–0.92) and the QOL-DAv2.0 was 0.96 (four scales range: 0.80–0.93). ICC of the SF-36v2 two components were 0.86 and 0.85 (eight scales range: 0.72–0.87) and the QOL-DAv2.0 was 0.94 (four scales range: 0.88–0.92). Convergent validity was lower between the two instruments (γ <0.70) while discriminant validity was acceptable within each instrument. Sensitivity was satisfied in self-evaluated health status (both instruments) and average daily methadone dose (SF-36v2 physical functioning and vitality scales; QOL-DAv2.0 except psychology scale). Responsiveness was acceptable in the improved health status change (SF-36v2 except vitality scale; QOL-DAv2.0 except psychology and symptoms scales) and deteriorated health status change (SF-36v2 except vitality, social functioning and mental health scales; QOL-DAv2.0 except society scale).

**Conclusions:**

The SF-36v2 and the QOL-DAv2.0 are valid tools and can be used independently or complementary according to different emphases of health-related quality of life evaluation in patients with MMT.

## Introduction

Drug abuse is a common problem over the past three decades in China [1]. Official statistics show that the number of registered drug users increased from 70,000 in 1990 [2] to 2.22 million at the end of May, 2013 [3]. Among drug users, about 75% to 85% depend on opioid [4]. Opioid dependence is a chronic maladaptive pattern of heroin or other opioid use, which often associated with co-morbid psychiatric disorders, and elevated risk of infection and transmission of blood-borne diseases (e.g., HIV/AIDS, hepatitis B or C), premature death and drug related crime [5]. It poses adverse effects for individuals and society and has become a major public health and social problem.

Methadone maintenance treatment (MMT) is a long term opioid replacement therapy with daily methadone administration [6]. In China, MMT was initiated as a pilot program in 8 clinics serving 1,029 drug users in 2004 [7] and subsequently expanded to 748 clinics serving 360,000 drug users in 2012 [8]. MMT bases on a harm reduction philosophy, represents one component of a continuum of treatment approaches for opioid dependent individuals, and allows a return-to-normal physiological, psychological and social functioning [9]. International evidence-based practices have proved that MMT is effective to prevent transmission of blood-borne diseases, to reduce illegal drug use and high-risk behaviors, to avoid criminal involvement and to enhance social productivity [10]. However, limited consideration is given to person-centered outcomes such as health-related quality of life (HRQoL) [11].

HRQoL is a multifactorial construct that describes the individual’s perceptions of physical, psychological and social functioning [12]. Unlike clinical parameters, HRQoL is a more holistic assessment of health status regarding the individual’s functional health and well-being, especially in chronic diseases [13]. Considering opioid dependence is a chronic disorder requiring a continuing care and support and MMT is a long term substitution therapy for opioid dependence [14], it is particularly important to use HRQoL as the primary endpoint for evaluating health and treatment effects in patients with MMT.

HRQoL is measured using generic or disease-specific instruments. Generic instruments provide a global assessment and allow for comparisons with other health conditions, while disease-specific instruments evaluate the difficulties presented in a specific group of patients or associated with a specific disorder [15]. In the area of opioid dependence research, the commonly used generic HRQoL instruments include the Brief Version of the World Health Organization Quality of Life Instrument (WHOQOL-BREF) [16]–[18], the EuroQol-5D (EQ-5D) [19], the Short Form 36 Health Survey (SF-36) [20]–[22] and the Lancashire Quality of Life Profile (LQoLP) [11], [23], and disease-specific instruments include the Injection Drug User Quality of Life Scale (IDUQoL) [24], the Health-related Quality of Life for Drug Abusers (HRQoLDA) [25] and the Quality of Life Scale for Drug Addicts (QOL-DAv2.0) [26], [27]. However, a dearth of study evaluated the Short Form 36 Health Survey version 2 (SF-36v2) in MMT patients and the complementary use of the SF-36v2 and the QOL-DAv2.0 has not been assessed in this population of mainland China.

The objective of this study was to evaluate reliability, validity, sensitivity and responsiveness of the SF-36v2 and the QOL-DAv2.0 in Chinese mainland MMT patients. To our knowledge, this is the first study to test psychometric properties of both instruments in the same population. Findings of this work will help find proper tools for health management and provide evidence for need-oriented use of the SF-36v2 and the QOL-DAv2.0 in HRQoL evaluation in patients with MMT.

## Methods

### Ethics Statement

The study protocol was reviewed and approved by the Human Research Ethics Committee of Xi’an Jiaotong University. The written informed consent was obtained from each recruited patient before the questionnaire survey.

### Subjects and Data Collection

The subjects were admitted patients of the Minle MMT clinic (privately funded) and the Xinan MMT clinic (publicly funded), each of which has the largest number of patients among the twelve MMT clinics in Xi’an, China. Inclusion criteria were aged 18 years or over and Chinese-speaking. If the patients had cognitive disorders or refused to give written informed consent, they were excluded.

Data were collected from March to September, 2012. The recruited MMT patients were given an individual face-to-face interview administered by the trained interviewers in a quiet and well-lit room. The patients answered the questions on sociodemographic and clinical characteristics, the SF-36v2 and the QOL-DAv2.0 at baseline (pretest). Besides, the two instruments were retested one week later and post-tested after 6-month follow-up, respectively.

### Data Measurement

#### The SF-36 health survey version 2 (SF-36v2)

The Chinese (simple) SF-36v2 was used in the study, which is provided by QualityMetric Incorporated [28]. It is the standard (4-week recall) form and consists of thirsty-six items. Except for the one single-item of self-evaluated transition (SET) (item 2), the scores of the other thirty-five items are summated into eight multi-item scales, including physical functioning (PF), limitations due to physical health problems [role-physical (RP)], bodily pain (BP), general health (GH), vitality (VT), social functioning (SF), limitations due to emotional health problems [role-emotional (RE)] and mental health (MH). The eight multi-item scales are aggregated into physical component summary (PCS) and mental component summary (MCS). The scoring of two summary components and eight scales was performed by QualityMetric Health Outcomes™ Scoring Software 4.0 based on norms with a mean of 50 and a standard deviation of 10 [29]. For all scales and summary components, higher scores demonstrate better HRQoL.

#### Quality of Life Scale for Drug Addicts (QOL-DAv2.0)

The QOL-DAv2.0 was developed by Wan et al. in 1995 [30]. It consists of forty items measuring four scales of physiology (PH) (9 items), psychology (PS) (9 items), society (SO) (11 items) and symptoms (ST) (11 items), and one independent single-item of self-evaluated health status (item 41). Each item is rated on a 5-point Likert scale (1 = none/very difficult/very poor to 5 = severe/very easy/very good) and ranges from 1 to 5. The four scale scores are calculated by the corresponding endorsed item scores, ranging from 9 to 45 (PH and PS) and 11 to 55 (SO and ST). The total score is calculated by the four scale scores, ranging from 40 to 200. For all scales and total score, higher scores indicate better HRQoL. Specifically, the single-item of self-evaluated health status (How do you evaluate your overall health status) has five levels (1 = very poor; 2 = poor; 3 = neither poor nor good; 4 = good; 5 = very good). In order to reflect the health change after follow-up, the patients were stratified into three health status, including improvement (i.e., the health status changed from very poor to poor, or very poor to good, and the like), status quo (i.e., the health status changed from very poor to very poor, or good to good, and the like), and deterioration (i.e., the health status changed from very good to good, or very good to poor, and the like).

### Data Analyses

A database was built using the software EpiData 3.1 and the data were double-entered by two different persons to capture data entry errors. All analyses were carried out using SPSS 13.0 (SPSS Inc., Chicago, IL, USA). A value of *P*<0.05 was considered as statistically significant.

Internal consistency reliability was measured by Cronbach’s α, with the value greater than 0.70 representing acceptable reliability [31]. Test-retest reliability was measured by intra-class correlation (ICC) between the one-week test-retest results. ICC ≤ 0.40 is considered poor to fair agreement, 0.41 to 0.60 moderate agreement, 0.61 to 0.80 good agreement, and >0.80 excellent agreement [32]. Floor and ceiling effects were calculated as the number and percentage of the total patients at the lowest and highest possible scores. This should be less than 15% regarding floor or ceiling effect respectively to ensure that the scales (SF-36v2 and QOL-DAv2.0), summary components (SF-36v2) and total score (QOL-DAv2.0) are capturing the full range of potential responses in MMT patients and that changes over time can be detected [33].

Convergent and discriminant validity were tested using multitrait-multimethod (MTMM) correlation matrix [15]. Convergent validity is determined by scale-to-scale correlations between different instruments, with higher value (γ ≥ 0.70) indicating the scales of different instruments measuring the same trait. Discriminant validity is determined by scale-to-scale correlations for each instrument, with lower value (γ <0.70) representing the scales measuring different traits. In this study, scale-to-scale correlations (spearman γ) were computed between the SF-36v2 eight scales and the QOL-DAv2.0 four scales.

Sensitivity was evaluated by ANOVA and relative efficiency (RE). RE was calculated as the ratio of *F*-statistics of the scales (SF-36v2 and QOL-DAv2.0), summary components (SF-36v2) and total score (QOL-DAv2.0), with the smallest *F*-statistics being the denominator in RE calculation. The smallest RE (RE = 1) represents the least sensitivity; the higher the RE value, the better the sensitivity [15]. In this study, the significant *F*-statistics with corresponding higher RE value represents acceptable sensitivity. Additionally, multiple linear regression analysis was used to further prove sensitivity of the SF-36v2 and the QOL-DAv2.0. The SF-36v2 two summary components and eight scales scores and the QOL-DAv2.0 four scales and total scores were dependent variables, respectively; independent variables were age, gender, education attainment, marital status, employment status, average monthly income over the past year (Chinese $), chronic disease, days of methadone intake and average daily dose of methadone intake (mg).

Based on stratification of the patients’ health status (i.e., improvement, status quo, deterioration), responsiveness was assessed by paired-samples *t*-test between the 6-month follow-up pre-post test results, with statistical significant mean differences representing magnitude observed change [34]. Another evaluation of responsiveness was standardized response mean (SRM), which is a ratio of observed change and the standard deviation reflecting variability of the change scores. SRM values of 0.20, 0.50, and 0.80 or greater have been proposed to represent small, moderate, and large responsiveness respectively [34]. In this study, the statistical significant mean difference with corresponding eligible SRM value demonstrates acceptable responsiveness.

## Results

A total of 1,212 patients were recruited at baseline, with 851 (70.2%) in the Minle MMT clinic and 361 (29.8%) in the Xinan MMT clinic. One hundred patients were randomly selected using a randomization code generated by computer software in the retest survey [35] and 1,010 patients completed the 6-month follow-up. Two-hundred and two (16.7%) patients lost to follow-up due to transferring to other MMT clinics (n = 153, 75.7%), being admitted to hospital (n = 40, 19.8%), or losing contact (n = 9, 4.5%). In the face-to-face interview, the patients understood the questions of the SF-36v2 and the QOL-DAv2.0 well and finished both questionnaires completely.

### Sociodemographic and Clinical Characteristics

The patients aged 42.48±6.24 (range: 21–65) years, with 934 (77.1%) males and 278 (22.9%) females. Education attainment included no schooling and primary (n = 141, 11.6%), secondary (n = 987, 81.4%) and tertiary (n = 84, 6.9%). Seven-hundred and five (58.2%) patients were married. Unemployed and employed patients accounted for 49.0% (n = 594) and 51.0% (n = 618) respectively. Average monthly income over the past year (Chinese $) of the patients were <1,000 (n = 708, 58.4%), 1,000–2,999 (n = 307, 25.3%) or ≥ 3,000 (n = 197, 16.3%). Six-hundred and sixteen (50.8%) patients had chronic disease. The patients had taken methadone for 717.15±435.57 (range: 1–1659) days since participated in MMT, with average daily methadone dose of 49.60±15.42 (range: 8–106) mg (Table 1).

**Table 1:** Sociodemographic and clinical characteristics of the patients (N = 1212).

Sociodemographic and clinical characteristics	*n*	%
Age (years)		
≤ 30	61	5.0
31–40	357	29.5
41–50	711	58.7
>50	83	6.8
Gender		
Male	934	77.1
Female	278	22.9
Education attainment		
No schooling and primary	141	11.6
Secondary	987	81.4
Tertiary	84	6.9
Marital status		
Single	229	18.9
Married	705	58.2
Divorced/Widowed	278	22.9
Employment status		
Unemployed	594	49.0
Employed	618	51.0
Average monthly income over thepast year (Chinese $)		
<1000	708	58.4
1000–2999	307	25.3
≥ 3000	197	16.3
Chronic disease		
Yes	616	50.8
No	596	49.2
Days of methadone intake		
≤ 90	69	5.7
91–180	93	7.7
181–365	170	14.0
>365	880	72.6
Average daily dose of methadone intake (mg)		
≤ 20	23	1.9
21–60	886	73.1
>60	303	25.0

### Reliability and Floor/ceiling Effect

Internal consistency reliability, test-retest reliability and floor/ceiling effect are detailed in Table 2. Cronbach’s α of the SF-36v2 physical and mental summary components were 0.80 and 0.86 (eight scales ranged from 0.73 to 0.92) and the QOL-DAv2.0 total score was 0.96 (four scales ranged from 0.80 to 0.93). ICC of the SF-36v2 two summary components were 0.86 and 0.85 (eight scales ranged from 0.72 to 0.87) and the QOL-DAv2.0 total score was 0.94 (four scales ranged from 0.88 to 0.92). Floor effects of the SF-36v2 and the QOL-DAv2.0 were all less than 15%. Except for the SF-36v2 physical functioning (18.2%), role-physical (23.9%), bodily pain (30.2%), social functioning (23.2%) and role-emotional (22.4%) scales, ceiling effects of the SF-36v2 remaining scales and summary components and the QOL-DAv2.0 four scales and total score were less than 15%.

**Table 2:** Internal consistency reliability, test-retest reliability, and floor/ceiling effect of the SF-36v2 and the QOL-DAv2.0 (N = 1212).

	Internal consistency reliability	Test-retest reliability†	Floor and ceiling effects [n (%)]‡	
	(Cronbach’s α)	(Intra-class correlation, ICC)	Floor effect	Ceiling effect
**SF-36v2**				
Physical component summary (PCS)	0.80	0.86	1 (0.1)	1 (0.1)
Mental component summary (MCS)	0.86	0.85	1 (0.1)	1 (0.1)
Physical functioning (PF)	0.88	0.73	1 (0.1)	**221 (18.2)**
Role-physical (RP)	0.92	0.75	51 (4.2)	**290 (23.9)**
Bodily pain (BP)	0.87	0.78	8 (0.7)	**366 (30.2)**
General health (GH)	0.78	0.87	38 (3.1)	2 (0.2)
Vitality (VT)	0.77	0.85	31 (2.6)	18 (1.5)
Social functioning (SF)	0.73	0.72	21 (1.7)	**281 (23.2)**
Role-emotional (RE)	0.91	0.79	65 (5.4)	**272 (22.4)**
Mental health (MH)	0.76	0.79	8 (0.7)	26 (2.1)
**QOL-DAv2.0**				
Physiology (PH)	0.88	0.88	1 (0.1)	5 (0.4)
Psychology (PS)	0.93	0.92	5 (0.4)	112 (9.2)
Society (SO)	0.80	0.89	1 (0.1)	1 (0.1)
Symptoms (ST)	0.92	0.90	1 (0.1)	118 (9.7)
Total score	0.96	0.94	2 (0.2)	1 (0.1)

† Test-retest reliability was assessed in a group of 100 patients.

‡ Floor and ceiling effects [n (%)]: the numbers and percentages of the patients at the lowest and highest possible scores.

Floor effect or ceiling effect ≥ 15% was in bold.

### Convergent and Discriminant Validity

All scale-to-scale correlation coefficients (γ) between the SF-36v2 and the QOL-DAv2.0 were less than 0.70. Except for the SF-36v2 role-emotional-to-role-physical correlation (γ = 0.75) and mental health-to-vitality correlation (γ = 0.73), and the QOL-DAv2.0 psychology-to-physiology correlation (γ = 0.75) and society-to-psychology correlation (γ = 0.74), the remaining scale-to-scale correlations within the SF-36v2 or the QOL-DAv2.0 were all less than 0.70 (Table 3).

**Table 3:** Convergent validity † and discriminant validity ‡ of the SF-36v2 and the QOL-DAv2.0: Multitrait-multimethod (MTMM) correlation matrix (spearman γ) (N = 1212).

	SF-36v2									QOL-DAv2.0			
	PF	RP	BP	GH	VT	SF		RE	MH	PH	PS	SO	ST
**SF-36v2**													
Physical functioning (PF)	1												
Role-physical (RP)	0.65	1											
Bodily pain (BP)	0.52	0.53	1										
General health (GH)	0.49	0.54	0.50	1									
Vitality (VT)	0.52	0.55	0.50	0.61	1								
Social functioning (SF)	0.47	0.58	0.56	0.52	0.54		1						
Role-emotional (RE)	0.53	**0.75**	0.50	0.49	0.52		0.59	1					
Mental health (MH)	0.46	0.54	0.53	0.53	**0.73**		0.59	0.61	1				
**QOL-DAv2.0**													
Physiology (PH)	0.53	0.57	0.56	0.64	0.66		0.56	0.57	0.63	1			
Psychology (PS)	0.46	0.51	0.51	0.55	0.59		0.56	0.55	0.65	**0.75**	1		
Society (SO)	0.42	0.44	0.47	0.53	0.49		0.52	0.46	0.54	0.65	**0.74**	1	
Symptoms (ST)	0.45	0.44	0.54	0.49	0.46		0.46	0.43	0.49	0.67	0.65	0.59	1

† Convergent validity: scale-to-scale correlations between the SF-36v2 and the QOL-DAv2.0.

‡ Discriminant validity: scale-to-scale correlations within the SF-36v2 or the QOL-DAv2.0.

The γ ≥ 0.70 was in bold.

### Sensitivity

Sensitivity was assessed in self-evaluated health status and average daily dose of methadone intake (mg). With respect to the five levels of health status, the SF-36v2 and the QOL-DAv2.0 scores increased from ‘very poor’ to ‘very good’ level by level with significant *F*-statistics (*P*<0.001). Compared with bodily pain scale which had the least sensitivity (RE = 1), other scales with better sensitivity were found in physiology (RE = 3.21) and general health (RE = 2.78) and the QOL-DAv2.0 total score (RE = 2.53). RE of the remaining scales and summary components ranged from 1 to 2, representing close sensitivity (Table 4). Based on controlling the influences of sociodemographic and clinical characteristics, multiple linear regression analysis further proved the differences in the SF-36v2 two summary components [physical component summary: unstandardized coefficient: 4.70, 95% confidence interval (CI): 4.17, 5.23; mental component summary: 6.23 (5.47, 6.99)] and eight scales [physical functioning: 3.52 (2.98, 4.06); role-physical: 5.61 (4.86, 6.35); bodily pain: 4.96 (4.17, 5.75); general health: 7.87 (7.18, 8.56); vitality: 6.49 (5.78, 7.21); social functioning: 5.02 (4.28, 5.76); role-emotional: 6.02 (5.14, 6.89); mental health: 5.86 (5.12, 6.60)], and the QOL-DAv2.0 four scales [physiology: 5.50 (5.06, 5.93); psychology: 5.02 (4.43, 5.62); society: 4.49 (3.97, 5.01); symptoms: 4.66 (4.00, 5.32)] and total score [19.67 (17.87, 21.47)] under the influence of self-evaluated health status with statistical significance (*P*<0.001). These data were not tabulated.

**Table 4:** Sensitivity of the SF-36v2 and the QOL-DAv2.0 in self-evaluated health status: scores (mean ± SD) and relative efficiency (RE) (N = 1212).

	Self-evaluated health status							
	Very poor	Poor	Neither poor nor good	Good	Very good	*F*	RE†	
	(n = 89)	(n = 280)	(n = 558)	(n = 246)	(n = 39)			
**SF-36v2**								
Physical component summary (PCS)	39.63±7.63	43.83±6.89	49.75±6.65	53.72±6.33	55.12±5.58	124.92**		**1.90**
Mental component summary (MCS)	30.10±10.73	35.55±9.87	41.84±9.34	47.57±8.29	52.24±7.54	100.18**		**1.52**
Physical functioning (PF)	41.74±8.68	46.41±7.31	50.95±6.34	53.35±5.58	54.55±5.10	78.75**		**1.20**
Role-physical (RP)	34.45±10.28	39.92±10.02	46.84±9.25	51.02±7.96	54.68±5.04	93.25**		**1.42**
Bodily pain (BP)	38.52±9.96	43.28±9.64	49.33±9.96	53.94±9.90	54.69±9.47	65.77** △		**1.00**
General health (GH)	28.03±8.02	32.09±7.85	40.59±8.67	48.63±9.93	53.15±8.41	182.72**		**2.78**
Vitality (VT)	35.44±9.60	41.15±8.75	47.88±8.67	53.92±8.82	57.09±9.73	120.81**		**1.84**
Social functioning (SF)	35.71±11.40	39.90±9.32	45.35±9.11	49.82±8.23	52.46±6.75	68.12**		**1.04**
Role-emotional (RE)	29.65±12.87	36.25±12.18	42.52±10.57	47.82±8.90	53.58±4.43	79.24**		**1.20**
Mental health (MH)	31.32±10.06	36.24±9.43	42.70±9.16	47.74±8.58	51.00±9.60	91.22**		**1.39**
**QOL-DAv2.0**								
Physiology (PH)	20.78±5.69	24.72±5.37	30.08±5.54	35.77±5.03	36.41±6.10	211.07**		**3.21**
Psychology (PS)	25.12±8.40	28.40±7.57	34.17±7.57	39.28±6.60	38.18±8.61	101.71**		**1.55**
Society (SO)	29.29±7.55	31.71±7.21	36.65±6.36	41.34±5.87	41.21±7.10	100.62**		**1.53**
Symptoms (ST)	35.16±9.71	40.15±8.55	45.03±8.11	49.33±6.85	49.44±8.90	74.91**		**1.14**
Total score	110.35±24.03	124.98±22.57	145.93±22.77	165.73±20.29	165.23±27.01	166.62**		**2.53**

† RE: relative efficiency was calculated as the ratio of *F*-statistics. The RE with significant *F*-statistics was in bold.

△ The scale with the smallest *F*-statistics was used as the denominator in RE calculation.

** *P*<0.01.

With respect to the three levels of average daily methadone dose (i.e., ≤ 20mg, 21–60mg, >60mg) [36], the SF-36v2 and the QOL-DAv2.0 scores decreased as the methadone dose increased. Regarding the comparison among the three group patients with different average daily methadone dose, significant *F*-statistics with higher RE value were found in the SF-36v2 physical functioning (RE = 3.14) and vitality (RE = 3.91) scales, and the QOL-DAv2.0 physiology (RE = 2.87), society (RE = 3.71) and symptoms (RE = 4.53) scales and total score (RE = 3.72), respectively (Table 5). Based on controlling the influences of sociodemographic and clinical characteristics, multiple linear regression analysis further proved the differences in the SF-36v2 vitality scale [−1.29 (−2.49, −0.09)] (*P* = 0.035) and the QOL-DAv2.0 symptoms scale [−1.39 (−2.45, −0.33)] (*P* = 0.01) with statistical significance under the influence of average daily methadone dose. These data were not tabulated.

**Table 5:** Sensitivity of the SF-36v2 and the QOL-DAv2.0 in average daily dose of methadone intake (mg): scores (mean ± SD) and relative efficiency (RE) (N = 1212).

	Average daily dose of methadone intake (mg)			*F*		RE†
	≤ 20 (n = 23)	21–60 (n = 886)	>60 (n = 303)			
**SF-36v2**						
Physical component summary (PCS)	51.31±5.43	48.74±7.82	48.07±8.40	2.15	1.82	
Mental component summary (MCS)	44.27±8.93	41.27±10.86	40.05±10.46	2.53	2.14	
Physical functioning (PF)	53.05±5.02	49.98±7.34	49.13±7.62	3.71*	**3.14**	
Role-physical (RP)	49.64±8.00	45.55±10.46	44.79±10.61	2.49	2.11	
Bodily pain (BP)	48.99±8.90	48.52±10.81	47.40±11.14	1.26	1.07	
General health (GH)	42.60±12.06	39.85±10.92	39.20±11.23	1.18△	1.00	
Vitality (VT)	51.82±8.27	47.19±10.30	45.80±10.80	4.61*	**3.91**	
Social functioning (SF)	47.31±9.07	44.62±10.00	44.00±10.33	1.33	1.13	
Role-emotional (RE)	43.76±11.82	41.88±12.09	40.45±11.73	2.00	1.69	
Mental health (MH)	45.30±8.86	41.86±10.54	40.80±10.33	2.57	2.18	
**QOL-DAv2.0**						
Physiology (PH)	32.96±5.88	29.58±6.96	29.07±7.40	3.39*	**2.87**	
Psychology (PS)	36.43±7.73	33.35±8.63	33.07±8.72	1.62	1.37	
Society (SO)	40.39±6.75	36.12±7.59	35.59±7.57	4.38*	**3.71**	
Symptoms (ST)	47.83±8.61	44.52±8.99	42.94±9.20	5.34**	**4.53**	
Total score	157.61±26.22	143.58±27.76	140.67±28.40	4.39*	**3.72**	

† RE: relative efficiency was calculated as the ratio of *F*-statistics. The RE with significant *F*-statistics was in bold.

△ The scale with the smallest *F*-statistics was used as the denominator in RE calculation.

* *P*<0.05. ** *P*<0.01.

### Responsiveness

According to health status changes, the patients were stratified into improvement, status quo and deterioration groups. In the improvement group (n = 33), significant mean differences were found in the SF-36v2 physical component summary and role-physical, general health, role-emotional and mental health scales and the QOL-DAv2.0 physiology scale; eligible SRM were found in the SF-36v2 except vitality scale and the QOL-DAv2.0 except psychology and symptoms scales. In the status quo group (n = 216), significant mean differences were found in the SF-36v2 mental component summary and general health and vitality scales and the QOL-DAv2.0 physiology and society scales; eligible SRM was found in the SF-36v2 general health scale. In the deterioration group (n = 761), significant mean differences were found in the SF-36v2 and the QOL-DAv2.0; eligible SRM were found in the SF-36v2 except vitality, social functioning and mental health scales and the QOL-DAv2.0 except society scale (Table 6).

**Table 6:** Responsiveness of the SF-36v2 and the QOL-DAv2.0: scores (mean ± SD) and standardized response mean (SRM) stratified on improvement, status quo, or deterioration in health status (N = 1010).

	Measurement of time		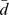	Sd	SRM	
	Pretest	Posttest				
Health status: improvement	n = 33	n = 33				
**SF-36v2**						
Physical component summary (PCS)	44.93±6.94	48.25±7.14	3.32*	8.72		**0.38**
Mental component summary (MCS)	34.64±12.85	38.69±6.50	4.05	14.15		**0.29**
Physical functioning (PF)	45.65±8.57	48.49±7.85	2.84	10.70		**0.27**
Role-physical (RP)	38.51±9.89	44.70±8.74	6.19**	12.60		**0.49**
Bodily pain (BP)	42.58±11.14	46.63±10.81	4.04	12.87		**0.31**
General health (GH)	36.89±8.52	40.39±6.79	3.50*	8.38		**0.42**
Vitality (VT)	44.41±10.97	45.22±8.17	0.81	13.33		0.06
Social functioning (SF)	39.41±11.21	42.30±10.18	2.89	13.38		**0.22**
Role-emotional (RE)	32.75±13.77	38.55±9.92	5.80*	15.85		**0.37**
Mental health (MH)	35.49±11.57	40.48±7.15	4.99*	12.94		**0.39**
**QOL-DAv2.0**						
Physiology (PH)	25.45±5.48	28.33±5.87	2.88*	6.47		**0.45**
Psychology (PS)	29.88±8.29	30.85±7.76	0.97	8.81		0.11
Society (SO)	31.61±7.44	34.03±7.09	2.42	7.62		**0.32**
Symptoms (ST)	40.64±8.91	41.88±10.10	1.24	11.29		0.11
Total score	127.58±24.27	135.09±26.51	7.52	24.41		**0.31**
**Health status: status quo**	n = 216	n = 216				
**SF-36v2**						
Physical component summary (PCS)	45.47±8.62	46.47±8.71	1.00	7.61		0.13
Mental component summary (MCS)	37.56±11.16	39.18±11.59	1.62*	10.64		0.15
Physical functioning (PF)	47.15±8.18	47.45±8.67	0.30	8.28		0.04
Role-physical (RP)	41.95±11.23	43.16±11.08	1.22	11.28		0.11
Bodily pain (BP)	44.85±11.17	46.06±11.82	1.21	11.87		0.10
General health (GH)	35.31±10.70	37.58±11.37	2.27**	8.52		**0.27**
Vitality (VT)	43.33±10.77	44.99±11.29	1.66*	10.19		0.16
Social functioning (SF)	41.63±11.15	43.09±10.50	1.46	11.11		0.13
Role-emotional (RE)	37.44±13.17	39.15±12.54	1.71	14.90		0.11
Mental health (MH)	38.58±11.07	39.54±11.66	0.97	10.50		0.09
**QOL-DAv2.0**						
Physiology (PH)	26.19±7.21	27.46±8.26	1.27**	6.59		0.19
Psychology (PS)	30.48±8.93	30.87±9.43	0.39	8.22		0.05
Society (SO)	33.72±7.99	34.93±8.54	1.21*	7.28		0.17
Symptoms (ST)	40.84±9.77	40.57±11.16	−0.27	9.74		−0.03
Total score	131.23±28.85	133.83±33.37	2.61	24.96		0.10
**Health status: deterioration**	n = 761	n = 761				
**SF-36v2**						
Physical component summary (PCS)	49.72±7.62	48.15±7.57	−1.57**	7.19		**−0.22**
Mental component summary (MCS)	42.62±10.13	40.54±10.35	−2.08**	10.05		**−0.21**
Physical functioning (PF)	50.55±7.00	49.49±7.25	−1.06**	7.29		**−0.15**
Role-physical (RP)	46.70±10.09	44.96±9.91	−1.74**	10.64		**−0.16**
Bodily pain (BP)	49.86±10.41	47.50±10.29	−2.36**	10.79		**−0.22**
General health (GH)	41.51±10.86	38.96±10.68	−2.55**	9.85		**−0.26**
Vitality (VT)	48.23±9.98	46.53±10.18	−1.69**	9.96		−0.17
Social functioning (SF)	45.94±9.45	44.60±9.58	−1.34**	10.09		−0.13
Role-emotional (RE)	43.23±11.23	40.64±11.60	−2.58**	12.26		**−0.21**
Mental health (MH)	43.09±9.96	41.18±10.20	−1.91**	10.00		−0.19
**QOL-DAv2.0**						
Physiology (PH)	30.85±6.71	29.05±7.03	−1.81**	5.79		**−0.31**
Psychology (PS)	34.56±8.30	32.93±8.67	−1.63**	7.82		**−0.21**
Society (SO)	37.18±7.27	36.06±7.87	−1.12**	6.66		−0.17
Symptoms (ST)	45.52±8.37	43.12±9.26	−2.40**	8.86		**−0.27**
Total score	148.11±26.39	141.16±28.61	−6.95**	23.23		**−0.30**

SD: standard deviation.

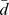
: mean difference between posttest score and pretest score.

Sd: standard deviation of the difference between posttest score and pretest score.

SRM: standardized response mean was calculated as 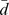/Sd. The SRM ≥ 0.20 was in bold.

Paired-samples *t*-test: * *P*<0.05. ** *P*<0.01.

## Discussion

This study demonstrated that the SF-36v2 and the QOL-DAv2.0 have satisfactory psychometric properties in patients with MMT. Internal consistency reliability (Cronbach’s α) of the SF-36v2 and the QOL-DAv2.0 were all above the critical threshold (0.70) for acceptable reliability, indicating that each questionnaire measures homogeneous extent in supporting the same concept [37]. Test-retest reliability of the SF-36v2 was good (ICC >0.60) and the QOL-DAv2.0 was excellent (ICC >0.80), showing the stability of each questionnaire over time [38]. The consistent result of the SF-36v2 reliability was also found by Lam et al. [39], who reported that Cronbach’s α of the eight scales ranged from 0.81 to 0.91 and ICC ranged from 0.54 to 0.93. With respect to the QOL-DAv2.0, Zhang et al. [40] found that Cronbach’s α of the four scales ranged from 0.88 to 0.93 and ICC ranged from 0.73 to 0.92. These consistent findings confirm that the SF-36v2 and the QOL-DAv2.0 have acceptable reliability.

Both the SF-36v2 and the QOL-DAv2.0 had eligible floor effects, with the lowest score percentages less than 15%. However, ceiling effects of the SF-36v2 were significant in physical functioning (18.2%), role-physical (23.9%), bodily pain (30.2%), social functioning (23.2%) and role-emotional (22.4%) scales, revealing that these five scale scores did not capture the full range of the corresponding responses and detect the special changes during the treatment process in patients with MMT [33]. Being a disease-specific instrument, the QOL-DAv2.0 had no significant ceiling effects in four scales and total score. It confirms that the QOL-DAv2.0 is superior to the SF-36v2 in capturing full range of changes in certain health domains in MMT patients.

Between the SF-36v2 and the QOL-DAv2.0, scale-to-scale correlation coefficients were all less than 0.70, indicating that the scales between the two instruments measuring different traits of HRQoL. HRQoL consists of physical, psychological and social aspects [15]. The SF-36v2 and the QOL-DAv2.0 reflect and measure these three aspects from generic and disease-specific viewpoints respectively; besides, the QOL-DAv2.0 is more specific in measuring the impacts of symptoms on HRQoL. Therefore, using the SF-36v2 and the QOL-DAv2.0 complementary is helpful to evaluate HRQoL comprehensively in patients with MMT.

For the SF-36v2 and the QOL-DAv2.0, scale-to-scale correlation coefficients within each instrument were less than 0.70, demonstrating that the scales within each instrument measuring different traits of HRQoL [15]. However, lower discriminant validity (γ ≥ 0.70) were found in the SF-36v2 role-emotional-to-role-physical correlation (γ = 0.75) and mental health-to-vitality correlation (γ = 0.73), and the QOL-DAv2.0 psychology-to-physiology correlation (γ = 0.75) and society-to-psychology correlation (γ = 0.74). The probable explanation may be that the items within these four-pair correlation scales relate to feelings or perceptions toward activities, function or psychological status under certain circumstances, which increase the correlation between the corresponding scales. Therefore, the discriminant validity of these four-pair scales needs further examination.

Sensitivity is the ability to detect differences between or among groups [15]. In patients with different self-evaluated health status, the SF-36v2 and the QOL-DAv2.0 were sensitive in detecting differences of all scales, summary components and total score, which were also proved by the results of multiple linear regression analysis. The finding indicates that both instruments were sensitive in detecting HRQoL score differences from the viewpoint of self-perception in health status.

In patients with different average daily methadone dose, both the SF-36v2 and the QOL-DAv2.0 scores decreased as the methadone dose increased, which is probably because of methadone adverse effects, including severe constipation, lethal cardiac complications, major depression, anxiety, and sexual dysfunction [41]. However, the two instruments were sensitive in specific scales. The SF-36v2 was mainly sensitive in physical functioning and vitality scales; after controlling for influences of sociodemographic and clinical characteristics, the multiple linear regression analysis further confirmed the difference in vitality scale. No significant *F*-statistics with corresponding eligible RE were found in the remaining two summary components and six scales. This is probably because of the following reasons: firstly, although the SF-36v2 scores decreased as the average daily methadone dose increased, there were no significant impacts of average daily methadone dose on the patients’ overall physical and mental health, limitations due to physical or emotional health problems, bodily pain, general health, and social function; secondly, the generic SF-36v2 measures health status in general population as well as specific disease population, which is poorer in detecting differences of special characteristics; thirdly, the sample sizes of patients with different average daily methadone dose were unbalanced (i.e., ≤ 20mg: n = 23; 21–60mg: n = 886; >60mg: n = 303), which may influence statistical inference results. Therefore, future work should further examine sensitivity of the SF-36v2 in MMT patients with different methadone dose.

Unlike the SF-36v2, the QOL-DAv2.0 was sensitive in physiology, society and symptoms scales and total score, especially in symptoms scale after controlling for influences of sociodemographic and clinical characteristics. The finding proves that the QOL-DAv2.0 is superior to the SF-36v2 in detecting differences in physical function, social function, symptoms and total health status in MMT patients with different average daily methadone dose. However, psychology scale had no significant *F*-statistics and eligible RE, which is probably because the patients’ psychological function was not significantly influenced by average daily methadone dose. Therefore, sensitivity of the QOL-DAv2.0 psychology scale needs further exploration.

Responsiveness is the ability of a scale to detect changes [15]. After the 6-month follow-up, both the SF-36v2 and the QOL-DAv2.0 were responsive in detecting improved and deteriorated health status changes. Regarding the improvement group, eligible SRM were found in the SF-36v2 and the QOL-DAv2.0, demonstrating that both instruments had better responsiveness. The scales without eligible SRM (i.e., the SF-36v2 vitality scale (0.06) and the QOL-DAv2.0 psychology (0.11) and symptoms (0.11) scales) indicate that the increased changes of the corresponding scores were inconsistent between patients [34], that is, some patients improved significantly in vitality, psychological function and symptoms, while others did not. On the other hand, the mean differences were not statistically significant in the SF-36v2 mental component summary and physical functioning, bodily pain, vitality and social functioning scales and the QOL-DAv2.0 except physiology scale, which is probably due to small sample size (n = 33). Therefore, a larger sample size is needed to confirm the responsiveness of these scales in future work.

Regarding the deterioration group, significant mean differences with eligible SRM were found in the SF-36v2 and the QOL-DAv2.0, suggesting that both instruments were responsive to detect decreased score changes in MMT patients after the 6-month follow-up. In MMT clinical practice, it is very helpful for clinicians to find those patients in such condition and to implement targeted interventions as early as possible. However, SRM of the SF-36v2 vitality (−0.17), social functioning (−0.13) and mental health (−0.19) scales and the QOL-DAv2.0 society (−0.17) scale were less than the critical value of 0.20, revealing the inconsistent decreased changes of the corresponding scores [34]. Therefore, it needs further examination on SRM of these scales in detecting deteriorated health status change in patients with MMT.

Specifically, significant mean difference with eligible SRM was found in the SF-36v2 general health scale in the status quo group, revealing discrepancy between the self-rated health status and corresponding real score difference. This is probably because the score change of general health was more significant than that of other health domains after the 6-month follow-up, which further proves better responsiveness of the SF-36v2 general health scale.

There were some limitations of the study. First, the SF-36v2 and the QOL-DAv2.0 were administered using a face-to-face interview, the performance of the instruments by self-completion will need to be confirmed by future work. Second, this study was conducted in Xi’an, which limited the generalization of the results to all of the Chinese mainland MMT patients.

The SF-36v2 and the QOL-DAv2.0 have been proved valid tools for assessing HRQoL in patients with MMT. Reliability of the two instruments was demonstrated to be strongly satisfactory. Convergent and discriminant validity showed that both instruments measured different traits of HRQoL. Better sensitivity was confirmed in both instruments in self-evaluated health status; regarding average daily methadone dose, the SF-36v2 was sensitive in physical functioning and vitality scales and the QOL-DAv2.0 in physiology, society and symptoms scales and total score. Both instruments were responsive in detecting improved and deteriorated health status changes. The SF-36v2 and the QOL-DAv2.0 can be used independently or complementary according to different emphases in HRQoL evaluation in patients with MMT.
