# Correction: Psychometrics of the Short Form 36 Health Survey Version 2 (SF-36v2) and the Quality of Life Scale for Drug Addicts (QOL-DAv2.0) in Chinese Mainland Patients with Methadone Maintenance Treatment

**DOI:** 10.1371/journal.pone.0089704

**Published:** 2014-02-26

**Authors:** The PLOS ONE Staff

Errors were introduced into Table 6 during the production process. The publisher apologizes for these errors. A corrected version can be viewed here.

**Table 6:** Responsiveness of the SF-36v2 and the QOL-DAv2.0: scores (mean ± SD) and standardized response mean (SRM) stratified on improvement, status quo, or deterioration in health status (N  =  1010).

	Measurement of time		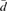	Sd	SRM
	Pretest Posttest				
**Health status: improvement**	n = 33	n = 33			
**SF-36v2**					
Physical component summary (PCS)	44.93 ± 6.94	48.25 ± 7.14	3.32*	8.72	**0.38**
Mental component summary (MCS)	34.64 ± 12.85	38.69 ± 6.50	4.05	14.15	**0.29**
Physical functioning (PF)	45.65 ± 8.57	48.49 ± 7.85	2.84	10.70	**0.27**
Role-physical (RP)	38.51 ± 9.89	44.70 ± 8.74	6.19**	12.60	**0.49**
Bodily pain (BP)	42.58 ± 11.14	46.63 ± 10.81	4.04	12.87	**0.31**
General health (GH)	36.89 ± 8.52	40.39 ± 6.79	3.50*	8.38	**0.42**
Vitality (VT)	44.41 ± 10.97	45.22 ± 8.17	0.81	13.33	0.06
Social functioning (SF)	39.41 ± 11.21	42.30 ± 10.18	2.89	13.38	**0.22**
Role-emotional (RE)	32.75 ± 13.77	38.55 ± 9.92	5.80*	15.85	**0.37**
Mental health (MH)	35.49 ± 11.57	40.48 ± 7.15	4.99*	12.94	**0.39**
**QOL-DAv2.0**					
Physiology (PH)	25.45 ± 5.48	28.33 ± 5.87	2.88*	6.47	**0.45**
Psychology (PS)	29.88 ± 8.29	30.85 ± 7.76	0.97	8.81	0.11
Society (SO)	31.61 ± 7.44	34.03 ± 7.09	2.42	7.62	**0.32**
Symptoms (ST)	40.64 ± 8.91	41.88 ± 10.10	1.24	11.29	0.11
Total score	127.58 ± 24.27	135.09 ± 26.51	7.52	24.41	**0.31**
**Health status: status quo**	n = 216	n = 216			
**SF-36v2**					
Physical component summary (PCS)	45.47 ± 8.62	46.47 ± 8.71	1.00	7.61	0.13
Mental component summary (MCS)	37.56 ± 11.16	39.18 ± 11.59	1.62*	10.64	0.15
Physical functioning (PF)	47.15 ± 8.18	47.45 ± 8.67	0.30	8.28	0.04
Role-physical (RP)	41.95 ± 11.23	43.16 ± 11.08	1.22	11.28	0.11
Bodily pain (BP)	44.85 ± 11.17	46.06 ± 11.82	1.21	11.87	0.10
General health (GH)	35.31 ± 10.70	37.58 ± 11.37	2.27**	8.52	**0.27**
Vitality (VT)	43.33 ± 10.77	44.99 ± 11.29	1.66*	10.19	0.16
Social functioning (SF)	41.63 ± 11.15	43.09 ± 10.50	1.46	11.11	0.13
Role-emotional (RE)	37.44 ± 13.17	39.15 ± 12.54	1.71	14.90	0.11
Mental health (MH)	38.58 ± 11.07	39.54 ± 11.66	0.97	10.50	0.09
**QOL-DAv2.0**					
Physiology (PH)	26.19 ± 7.21	27.46 ± 8.26	1.27**	6.59	0.19
Psychology (PS)	30.48 ± 8.93	30.87 ± 9.43	0.39	8.22	0.05
Society (SO)	33.72 ± 7.99	34.93 ± 8.54	1.21*	7.28	0.17
Symptoms (ST)	40.84 ± 9.77	40.57 ± 11.16	-0.27	9.74	-0.03
Total score	131.23 ± 28.85	133.83 ± 33.37	2.61	24.96	0.10
**Health status: deterioration**	n = 761	n = 761			
**SF-36v2**					
Physical component summary (PCS)	49.72 ± 7.62	48.15 ± 7.57	-1.57**	7.19	**-0.22**
Mental component summary (MCS)	42.62 ± 10.13	40.54 ± 10.35	-2.08**	10.05	**-0.21**
Physical functioning (PF)	50.55 ± 7.00	49.49 ± 7.25	-1.06**	7.29	**-0.15**
Role-physical (RP)	46.70 ± 10.09	44.96 ± 9.91	-1.74**	10.64	**-0.16**
Bodily pain (BP)	49.86 ± 10.41	47.50 ± 10.29	-2.36**	10.79	**-0.22**
General health (GH)	41.51 ± 10.86	38.96 ± 10.68	-2.55**	9.85	**-0.26**
Vitality (VT)	48.23 ± 9.98	46.53 ± 10.18	-1.69**	9.96	-0.17
Social functioning (SF)	45.94 ± 9.45	44.60 ± 9.58	-1.34**	10.09	-0.13
Role-emotional (RE)	43.23 ± 11.23	40.64 ± 11.60	-2.58**	12.26	**-0.21**
Mental health (MH)	43.09 ± 9.96	41.18 ± 10.20	-1.91**	10.00	-0.19
**QOL-DAv2.0**					
Physiology (PH)	30.85 ± 6.71	29.05 ± 7.03	-1.81**	5.79	**-0.31**
Psychology (PS)	34.56 ± 8.30	32.93 ± 8.67	-1.63**	7.82	**-0.21**
Society (SO)	37.18 ± 7.27	36.06 ± 7.87	-1.12**	6.66	-0.17
Symptoms (ST)	45.52 ± 8.37	43.12 ± 9.26	-2.40**	8.86	**-0.27**
Total score	148.11 ± 26.39	141.16 ± 28.61	-6.95**	23.23	**-0.30**

SD: standard deviation.

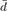
: mean difference between posttest score and pretest score.

Sd: standard deviation of the difference between posttest score and pretest score.

SRM: standardized response mean was calculated as 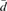/Sd. The SRM ≥ 0.20 was in bold.

Paired-samples *t*-test: * *P* < 0.05. ** *P* < 0.01.
